# Long-term results of uncemented allograft prosthesis composite reconstruction for the tumor in proximal femur: a minimum follow-up of sixty-five months

**DOI:** 10.1186/s12891-021-03991-6

**Published:** 2021-02-01

**Authors:** Cai Liu, Li Min, Yong Zhou, Yi Luo, Fan Tang, Minxun Lu, Hong Duan, Wenli Zhang, Xinzhu Yu, Chongqi Tu

**Affiliations:** Department of Orthopedic Surgery, West China Hospital of Sichuan University, Guoxue Road 37#, Chengdu, 610041 China

**Keywords:** Uncemented, Allograft–prosthesis composite, Proximal femur, Tumor

## Abstract

**Background:**

Uncemented allograft prosthesis composite (APC) has been applied for tumorous bone defect reconstruction in the proximal femur. However, the long-term results are rarely reported. This study aimed to evaluate long-term outcomes of uncemented APC.

**Methods:**

Eighteen patients who received uncemented APC reconstruction in the proximal femur after tumor resections were retrospectively reviewed.

**Results:**

The average resection length was 110 mm (80–154) and the average follow-up was 106.7 months (65–141). Bone union achieved in all patients with an average duration of 7.6 months (5–10). The average HHS, MSTS score and gluteus medius strength at one-year follow-up were 88.0 (80–94), 25.2 (22–28) and 4 (3–5), respectively. While at the last follow-up, the HHS, MSTS score and gluteus medius strength were 83.0 (48–100), 24.0 (10–30) and 4 (2–5), respectively. Five intraoperative fractures were fixed with cerclage wires. Two postoperative periprosthetic and prosthetic fractures received a revision. Three local recurrent patients received a secondary surgery. One of these three lung metastatic patients underwent lung metastatic tumor resection. Another two patients were diagnosed with both bone and lung metastases, only one of them underwent amputation. Two greater trochanteric fractures received no treatment. There were10 severe, 3 moderate and 5 mild allograft resorptions without treatment.

**Conclusion:**

Uncemented APC is a reliable reconstruction for neoplastic bone defect of the proximal femur, especially for the young patient who expected long-life expectancy and good function. Though allograft resorption and trochanteric fracture are the common complications, they seem no effect on the function.

**Supplementary Information:**

The online version contains supplementary material available at 10.1186/s12891-021-03991-6.

## Background

The proximal femur is one of the most frequent sites for aggressive benign tumors and primary malignancies [1]. Functional reconstructions for oncological bone defects in the proximal femur remain challenging. Since the 1960s, reconstruction with osteoarticular allograft has been used [2, 3]. However, the limitation has been reported as the high rate (nearly 70%) of complications, such as fracture, infection, degeneration and disintegration [4, 5]. At present, reconstruction with endoprosthesis, known as the gold standard, still has its shortcomings, including lower abductor muscle strength, impaired function, Trendelenburg gait, loosening, structural failure and dislocation [6–9]. Meanwhile, with the development of neoadjuvant chemotherapy, radio-therapy, immunotherapy and targeted therapy, more and more patients with bone malignancies tend to have a better prognosis with longer life expectancy [10]. Hence, it’s of great significance to preserve favorable limb function and increase implant survival after reconstruction.

Allograft prosthesis composite (APC), a hybrid of endoprosthesis and allograft, holds the merits of these two methods with fewer disadvantages [11]. Since the 1980s, it has been used for the reconstruction of neoplastic bone defects and revision hip arthroplasty (RHA), especially when the circumferential femoral bone defects are greater than 3 cm in length [12–14]. Compared with endoprosthesis, APC reconstruction seems biological based on the advantages of improving gait, restoring bone stock, biological reconstruction of the abductors, providing weight-bearing and reducing stress shielding after bone union [1, 15, 16]. Nowadays, the APC technique can be divided into cemented, uncemented or partial cemented. However, the cemented or partial cemented APC hold a high rate of nonunion in previous studies, up to 23% [1, 14, 17–21]. Additionally, revision of cemented or partial cemented APC needs to remove the allograft, which makes the advantage of restoring bone stock no longer exist [14, 21, 22].

Since 2007, uncemented APC has been used for the reconstruction of tumorous proximal femoral defect in our institute [23]. The short-term follow-up was promising [23]. After a long-term follow-up of these patients, some new information is worth sharing. Thus, the objectives of our study were to: 1) present the long-term clinical, oncological outcomes and complications of uncemented APC reconstruction; 2) how to manage the complications.

## Methods

From 2007 to 2014, we have performed uncemented APC reconstruction for 25 cases with a malignant or aggressive benign bone tumor in the proximal femur. Indications have been described in our previous study [20, 23]. In this study, exclusion criteria were: 1) metastasis of the proximal femur; 2) the malignant tumor invaded the hip joint, even the acetabulum; 3) the follow-up less than 5 years. Therefore, 7 patients were excluded, including 1 infection, 1 death and 5 lost to follow-up. In all, 18 patients (7 male and 11 female, sex ratio: 0.6, 18 hips) were analyzed in this study. The average age was 30.8 years (17–52). The pathological diagnoses were 2 chondrosarcoma (CS), 1 malignant fibrous histiocytoma (MFH), 8 giant cell tumor of bone (GCTB), 3 osteoblastoma (OB), 1 osteoblastic osteosarcoma (OBOS), 2 osteosarcoma (OS) and 1 fibroblastic osteosarcoma (FBOS). Eleven benign tumors were grade 3 according to the Enneking stage [24]. For malignant tumors, there were 6 grade IIB and 1 grade IIA according to the American Joint Committee on Cancer (AJCC) Staging system [25]. Seven patients had operations before, including 4 GCTB, 1 FBOS and 2 OB. Eight patients presented with pathological fractures, including 5 GCTB, 2 OS and 1 CS (Table 1). Five patients had neoadjuvant chemotherapy.

**Table 1:** Patients characteristics^a^

Data	
**Age (years)**	30.8 (17 to 52)
**Gender**	
Male	7 (38.9%)
Female	11 (61.1%)
**Diagnosis**	
Chondrosarcoma	2 (11.1%)
Malignant fibrous histiocytoma	1 (5.6%)
Giant cell tumor of bone	8 (44.4%)
Osteoblastoma	3 (16.7%)
Osteoblastic osteosarcoma	1 (5.6%)
Osteosarcoma	2 (11.1%)
Fibroblastic osteosarcoma	1 (5.6%)
**Primary**	18 (100%)
**Recurrence before surgery**	7 (38.9%)
**Pathological fracture before surgery**	
Yes	8 (44.4%)
No	10 (55.6%)
**Chemotherapy**	5 (27.8%)
**Resection length (mm)**	110 (80 to 154)
**Acetabular type**	
DePuy metal socket	17 (94.4%)
Cage	1 (5.6%)
**Union time of the host and allograft (months)**	7.6 months (5 to 10)

^a^Values are numbers of patients (percentages) unless otherwise indicated

### Preoperative assessments

The examinations of 100% magnified X-ray (Whole femur and pelvic), computed tomography (CT) scan, magnetic resonance imaging (MRI) and single-photon emission computed tomography (SPECT) were necessary. According to these results, we identified the resection length, the exact boundary of the tumor, the narrowest diameter of the medullary canal and cortex thickness of the distal femur, which were beneficial for choosing the optimum allograft. All diagnoses were confirmed by biopsies or ex-surgeries. Neoadjuvant chemotherapies were routinely taken for patients with malignant bone tumors. All the patients were informed of the use of allografts. The allografts were obtained from the bone bank of Sichuan province, People’s Republic of China.

### Surgical techniques

All the operations were performed with a lateral approach by the senior surgeon (Chongqi Tu) in West China Hospital. The previous biopsy tracks were removed en bloc. The surgical procedure included 3 major steps: allograft preparing, tumor resection and reconstruction, which had been described thoroughly in our previous study [23].

The allograft preparation was done on another sterile operation table, including 3 procedures: First, the allograft was soaked with povidone-iodine solution for 30 mins and pulsatile lavaged with a large amount of saline solution and degreased with medical alcohol; Second, the bone was resected the exact length according to the measurements before the surgery through the imageology data and during the operation; Finally, the greater and lesser trochanters were prepared by removal of the allograft tissue and drilled for the reconstruction of the soft tissue.

The tumor resection was performed on the operation table. The abductor muscles (Mainly gluteus medius) and iliopsoas were saved as much as possible and marked respectively. To achieve a wide, safe boundary, an en bloc resection with at least 2 cm was performed for aggressive benign or bordering tumors and a 5 cm for primary malignant tumors. No greater trochanteric osteotomy was performed in our series. Before osteotomy, the precise resection length was measured again, which was measured from the tip of the greater trochanter. If possible, a 10-15 mm normal periosteal cuff was preserved. Then, the osteotomy was carried out horizontally and the distal femoral medullary tissue was sent for frozen biopsy to make sure the en bloc resection.

Reconstruction proceeded as follows: Initially, osteotomy of the neck was performed and the medullary canal was reamed to fit the prosthesis. A propriate length of prosthesis was selected to meet the ratio of prosthetic length in host bone to in the allograft was close to (1–1.5):1. After a trial fitting, the prosthesis was fixed into the allograft bone by press-fit. Then, a second trial was performed and the host bone was reamed to fit the composite prosthesis. After that, the allograft composite was inserted into the host bone by pressure, similar to an endoprosthesis. To make sure the prosthetic anteversion was around 15°, the knee was flexed at an angle of around 90°. Then, the allograft composite would be gradually inserted into the host with an angle around 105° formed by comparing the tibial axis with the prosthetic femoral neck axis. Once the allograft and host bone contacted well, the junction was covered with granular allogenous spongy bone and the periosteal cuff. Finally, the important muscles were reconstructed. The gluteus medius and gluteus minimus were fixed together to the greater trochanter in 13 patients, while the rest patients with the gluteus medius insertion preserved, sutured onto the allograft greater trochanter, which had been described in our previous studies [20, 23]. Meanwhile, the iliopsoas was sutured to the lesser trochanter of the allograft. The gluteus maximus tendon was sutured to the tissues surrounded instead of its anatomic place. All patients had the vastus lateralis, the gluteus medius and the fascia lata sutured together.

### Postoperative management

The prophylactic intravenous antibiotics were used for 1–2 days as usual. An abduction T-shaped pillow and anti-rotation shoes were used to ensure the lower extremity in the abduction-neutral position without rotation of the affected limb. The isometric exercises of quadriceps femoris, moves of the ankle, raise of buttock, the practice of cough and deep breath were recommended immediately after the operation and last for 4 weeks in bed. Antithrombotic drugs were used in this period until partial weight-bearing was admitted. Then, 8 weeks later, full weight-bearing was allowed.

### Clinical and radiographic assessments

All patients were followed up in the outpatient clinic monthly in the first 6 months, after that, every 3 months for the first 2 years and then annually. Harris Hip Score (HHS: for which a score of < 70 is poor, 70–79 is fair, 80–89 is good and 90–100 is excellent) and Musculoskeletal Tumor Society (MSTS) score were used for the evaluation of the functional outcomes. To assess the impact of postoperative complications on function, the HHS and MSTS score of the pre-operation, one-year follow-up and last follow-up were recorded, respectively [26, 27]. The abductors’ strength was scored by the strength of the gluteus medius.

Whole femoral and 100% magnified AP pelvis X-rays were performed at 1, 3, 6, 9 and 12 months after surgery, then, annually. In the later follow-up, Tomosynthesis-Shimadzu metal artifact reduction technology (T-SMART) or CT would be taken if necessary, especially when the follow-up was over 5 years. Bone union at the host and allograft junction was defined as blurring showed in the radiography, usually combined with bridging trabeculae at the junction without radiolucent lines. Nonunion was confirmed if no further progress at the conjunction was observed for more than 1 year [23].

The allograft and the host were divided into 7 zones similar to those of Gruen [28]. The resorption in these zones was recorded. The severity of the resorption was graded as mild, moderate and severe [29].

### Statistical analysis

Continuous variables were expressed as mean (Range) and compared using the Wilcoxon Signed Ranks Test and Mann-Whitney Test. *P* < 0.05 was considered to be significant. Statistical analysis was performed with SPSS Advanced Statistics 23.0 software (IBM, Armonk, NY).

## Results

The average resection length was 110 mm (80–154) and the average follow-up was 106.7 months (65–141). Bone union at the allograft-host junction achieved in all patients on an average of 7.6 months (5–10) without a delayed union.

The HHS was significantly improved from 41.0 (20–58) pre-operation to 88.0 (80–94) at one-year follow-up (*p* < 0.01) while 83.0 (52–100) at the last follow-up (*p* < 0.01). The MSTS score was improved from 9.2 (4–15) pre-operation to 25.2 (22–28) at one-year follow-up (p < 0.01) while 24.0 (10–30) at the last follow-up (p < 0.01). According to the HHS, at one-year follow-up, 9 (50%) patients got excellent and 9 (50%) got good. However, 11 (61.1%) patients got excellent, 2 (11.1%) achieved good, 1 (5.6%) fair and 4 (22.2%) poor at the last follow-up. The average strength of the gluteus medius was 4 (3–5) at one-year follow-up while 4 (2–5) at the last follow-up (*p* = 0.700) (Table 2).

**Table 2:** Demographic characters and outcomes of 18 patients

P	A/G	Diag	St(E/A)	RL (mm)	Follow-up (months)	HHS (Pre)	HHS1	HHS2	MSTS (Pre)	MSTS1	MSTS2	SGM	Complications	Treatment
1	20/F	CS	IIB/IIB	146	141	47	90	82	10	26	24	3	Resorption (Gruen1.2.7)	–
2	35/F	MFH	IIA/IIB	146	135	43	86	93	9	24	27	5	Resorption (Gruen1.7)	–
3	31/F	GCTB	3/−	86	134	54	90	90	14	26	26	4	Resorption (Gruen1.7)	–
4	23/M	OB	3/−	97	134	58	80	88	15	23	25	4	Resorption (Gruen1.7), IHF	–
5	39/F	GCTB	3/−	80	126	47	85	95	10	24	27	5	Resorption (Gruen1.7)	Cerclage wires
6	25/F	GCTB	3/−	82	122	30	94	92	6	28	27	5	Resorption (Gruen1.7)	–
7	17/F	OB	3/−	94	122	54	88	94	14	25	28	5	Resorption (Gruen1.7)	–
8	47/M	OBOS	IIA/IIA	100	112	55	80	94	14	23	28	5	Resorption (Gruen1.7), IAF	Cerclage wires
9	17/F	OB	3/−	131	110	54	94	53	14	28	13	2	Resorption (Gruen1.2.7), LR, GTF (P), metastasis of distal femur and lung	Hemipelvectomy
10	45/M	OS	IIB/IIB	127	107	20	88	100	4	25	30	5	Resorption (Gruen1.2.7), IHF	Cerclage wires
11	52/F	CS	IIB/IIB	123	107	20	80	91	4	23	26	4	Resorption (Gruen1.7)	–
12	40/F	GCTB	3/−	93	105	30	90	92	6	26	27	4	Resorption (Gruen1.7), SF (P)	Change the stem
13	17/M	OS	IIB/IIB	130	96	20	90	100	5	26	30	5	Resorption (Gruen1.2.7), GTF (P), metastasis of lung	Metastasis resection
14	25/M	GCTB	3/−	80	88	30	92	94	6	27	28	4	Resorption (Gruen1.7)	–
15	33/M	GCTB	3/−	135	75	42	92	52	8	27	16	2	Resorption (Gruen1.2.7), SF (P), LR	Endoprosthesis, recurrence resection
16	43/M	GCTB	3/−	98	71	32	86	65	6	24	19	3	Resorption (Gruen1.7), IAF, LR	Cerclage wires, recurrence resection
17	27/F	GCTB	3/−	83	70	47	80	48	10	22	10	4	Resorption (Gruen1.7), IAF, metastasis of ipsilateral pelvic and lung	Cerclage wires
18	24/F	FBOS	IIA/IIB	154	65	48	90	71	10	26	21	2	Resorption (Gruen1.7)	–

*Abbreviations*: *P* Patient number, *A/G* Age(years)/gender, *F* Female, *M* Male, *Diag* Diagnosis, *CS* Chondrosarcoma, *MFH* Malignant Fibrous Histiocytoma, *GCTB* Giant Cell Tumor of Bone, *OB* Osteoblastoma, *OBOS* Osteoblastic Osteosarcoma, *FBOS* Fibroblastic Osteosarcoma, *St(E/A)* Ttage(Enneking/AJCC), *Pa* Pathological fracture, *Re* Recurrence, *RL* Resection length, *HHS* Harris Hip Score, *Pre* Pre-operation, *HHS1* HHS(One-year Follow-up), *HHS2* HHS(One-year Follow-up), *MSTS* Musculoskeletal Tumor Society, *MSTS1* MSTS (One-year follow-up), *MSTS2* MSTS (Last Follow-up), *SGM* Strength of gluteus medius, *IHF* Intra-operative host fracture, *GTF* Greater Trochanteric Fracture, *IAF* Intra-operative allograft fracture, *LR* Local recurrence, *SF* Stem fracture, *P* Post-operation

**Fig. 1:**
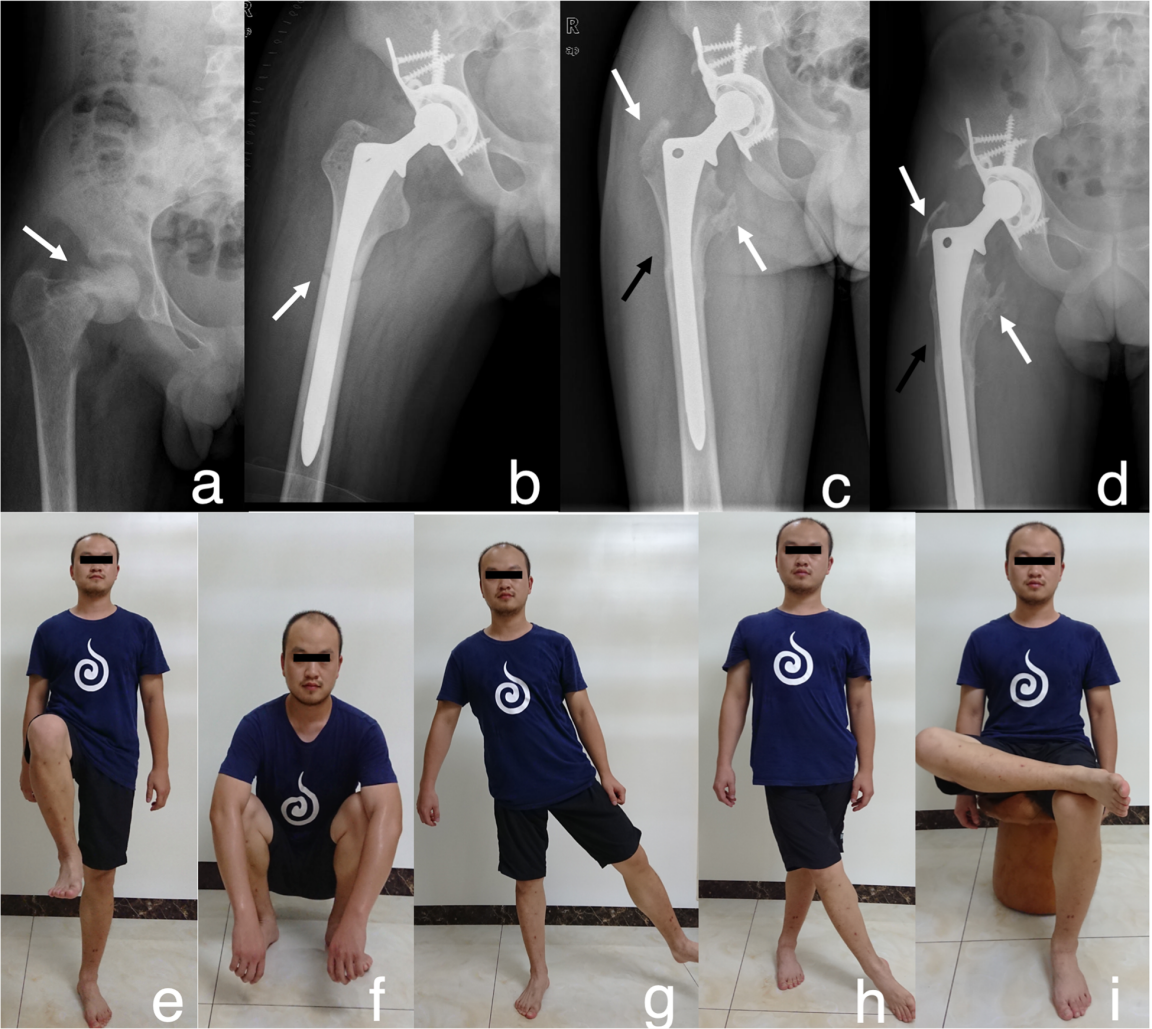
Patient (No. 13) with the diagnosis of OS in the right femoral neck. **a** White arrow indicated pathological fracture occurred during the chemotherapy pre-operation. **b** The immediate radiograph after the reconstruction with uncemented APC. **c**-**d** 12 months and 96 months post-operation: white arrows indicated the avulsion of the trochanteric fractures, while the black arrow indicated the resorption of the allograft bone. **e**-**i** The function of the hip, 96 months post-operation

There were 5 intraoperative fractures, 2 postoperative periprosthetic and prosthetic fractures (PPPFs), and 2 trochanteric fractures. All intraoperative fractures were longitudinal, no more than 2 cm in length, stable and incomplete crack fractures, which were fixed with cerclage wires. One patient had a recurrent in the area of the allograft (Patient No.16). The follow-up X-ray of the rest 4 showed the union of the allograft-host junction, no subsidence of the prosthesis and no obvious lucent line between the prosthesis and allograft. Two PPPFs received a revision. One (Patient No.12) occurred at 10 mm above the allograft-host junction line 105 months postoperatively. A longer stem was used without removing the allograft. The other one (Patient No.15) with soft tissue recurrence, occurred at 37 mm above the allograft-host junction line 75 months postoperatively. The revision with endoprosthesis and recurrent tumor resection was carried out (Table 2). Two patients who had a greater trochanteric fracture 6 months post-operation received no treatment (Fig. 1).

**Fig. 2:**
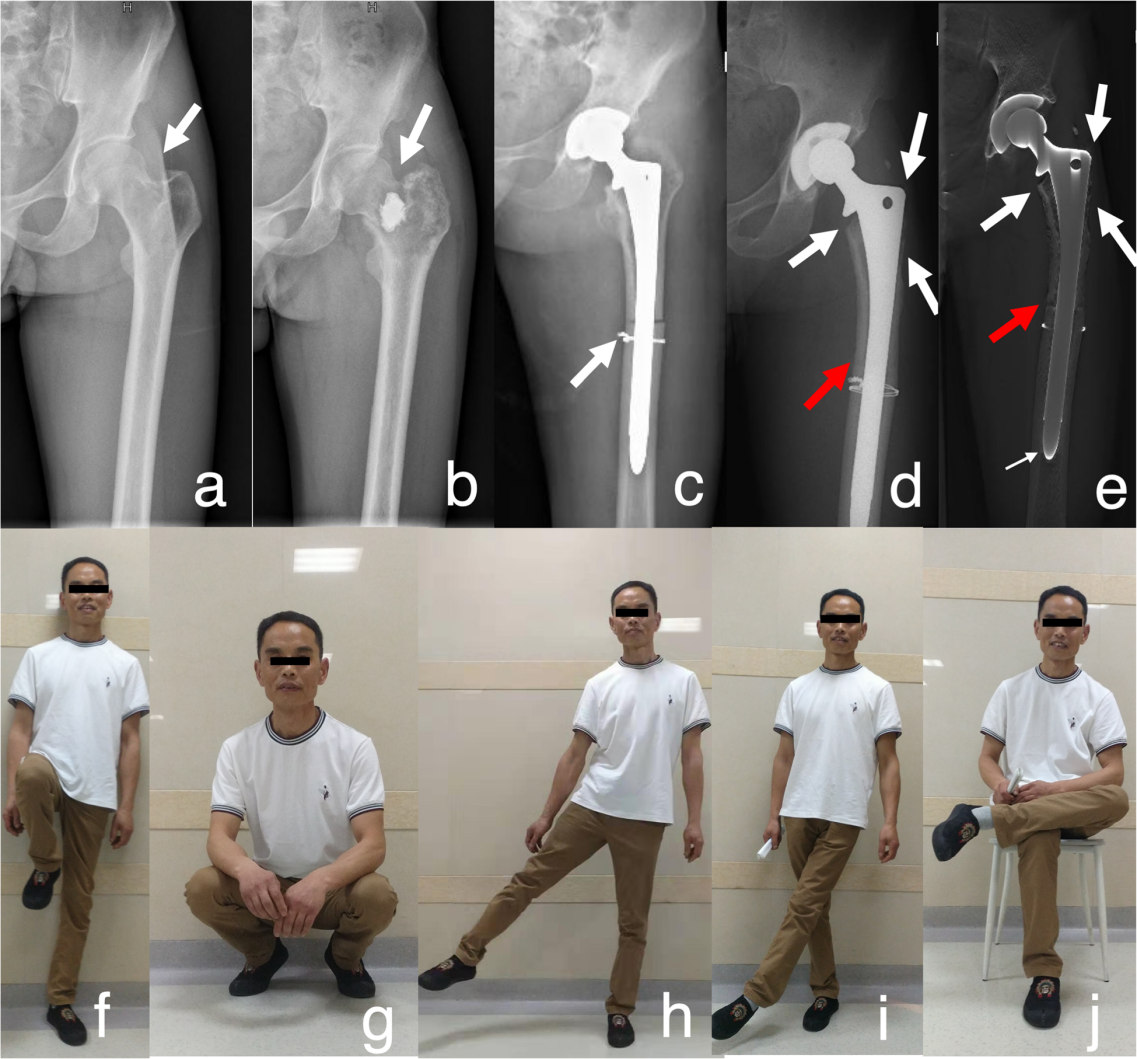
Patient (No.10) with the diagnosis of OS of the left femoral neck. **a** The white arrow indicated the lesion. **b** The white arrow indicated the pathological fracture occurred after the incisional biopsy during chemotherapy. **c** Immediate radiograph after the surgery of uncemented APC, the white arrow expressed intraoperative fracture and fixed with cerclage wires. **d**-**e** The last follow up radiograph and Tomosynthesis-Shimadzu metal artifact reduction technology (T-SMART) 107 months post-operation: the thick white arrows showed the resorption of the Gruen 1, Gruen2 and Gruen 7, the red arrows indicated bone union at the junction, the thin white arrows showed bone growing around the distal stem. (f-j) The function of the hip, 107 months post-operation

Seven severe, 6 moderate and 3 mild resorptions were identified at one-year follow-up. At the last follow-up, there were 10 severe, 3 moderate and 5 mild resorptions. All involved in Gruen 1, 2 and 7 zone (Fig. 2). No treatment was performed.

Local recurrence (LR) developed in 3 patients. One patient (Patient No. 9) underwent a hemipelvectomy after several operations as the LR invaded the ipsilateral pelvic, and metastases in the lung and distal femur. One (Patient No.16) received the resection of the greater trochanteric for LR 71 months after the last operation. The last one (Patient No.15) has been mentioned above. Three patients developed lung metastasis. One (Patient No.13) received the metastasis removal 1 year post-operation. One (Patient No.17) was observed metastases in the ipsilateral pelvic and lung 9 months post-operation, having no treatment. The last one (Patient No. 9) has been mentioned above.

## Discussion

Up to now, uncemented APC reconstruction for proximal femoral defect has only been reported with minor sample and short-term follow-up (Table 3). In our series, the clinical outcomes were updated with longer follow-up and enlarged samples based on our previous study regarding uncemented APC [23].

**Table 3:** Literature review of Allografts Prosthesis Composite for the tumoral skeletal defects in proximal femur

References	No	Reasons		Folllow-up (months)	Type of Acetabulum		Union time (months)	Type of fixation				OR (%)	Complications						LS (%)	Assessment	
		Tu	RHA		THA	Bi		CA	UCA	PCA	AP		AL	SF	IN	TP/D	Non	Ins		HHS	MSTS (%)
Gitelis [12]	11	11	/	/	/	/	/	7	/	4	4	/	/	/	/	/	/	/	/	/	/
Zehr [13]	18	18	/	63,2 (9–122)	11	7	13.5 (12–15)	14	2	2	2	/	/	/	3	/	1	/	94	/	77.7 (67–83)
McGoveran [11]	16	16	/	47 (24–93)	/	/	/	5	/	11	2	43.8	/	3	3	/	2	/	100	/	58.3
Donati [30]	27	27	/	58 (11–126)	3	24	7 (3–12)	4	/	23	/	18.2	/	1	1	5	1	/	100	/	92 (75–100)
Langlais [15]	21	21	/	72	/	/	/	21	/	/	/	38	4	/	/	10	4	/	100	/	76.9 (43–97)
Farid [31]	20	20	/	77.5 (24–335)	/	/	/	18	/	2	/	40	/	/	1	8	2	2	100	/	82
Lee [16]	15	1	14	50.4 (24–117.6)	15	/	11.6 (4–16)	3	/	12	/	26.7	2	/	1	/	2	1	/	83.2 (67–96)	/
Biau [17]	32	32	/	68 (2–232)	22	10	/	32	/	/	/	28.1	1	3	4	1	/	/	100	/	/
Muscolo [18]	38	26	12	90 (36–204)	38	/	/	/	/	38	38	29	/	7	3	1	/	/	96	/	90 (43.3–100)
Malhotra [19]	18	18	/	54 (18–79)	18	/	6.8 (4.5–11)	/	/	18	/	/	/	/	/	/	/	/	100	90.3 (84–96)	/
Min [23]	12	12	/	24 (16–35)	7	5	(5–9)	/	12	/	/	/	/	/	/	/	/	/	100	81.6 (66.2–92.7)	87.3 (80–96.7)
Min [20]	28	28	/	50.7 (15–138)	17	11	(9–18)	28	/	/	/	3.6	/	2	/	/	6	/	100	80.6 (66.2–92.7)	88.3 (70–96.7)
Dubor y[1]	46	32	14	176.4 (75.6–391.2)	32	14	/	42	2	2	/	/	4	6	3	5	7	4	/	/	77 (50–96.7)
Current series	18	18	/	106.7 (65–141)	18	/	7.6 (5–10)	/	18	/	/	22.2	/	2	/	3	/	/	94.4	83 (48–100)	80 (33.3–100)

*Abbreviations*: *No* Number, *Tu* Tumor, *RHA* Revision of hip arthroplasty, *THA* Total hip arthroplasty, *Bi* Bipolar, *CA* Cemented allograft composite, *UCA* Uncemented allograft composite, *PCA* Partial cemented allograft composite, *AP* Additional plate, *OR* Overall revision rate, *AL* Aseptic loosening, *SF* Structural failure, *IN* Infection, *TP* Tumor progressing, *D* Death, *Non* Non-union, *Ins* Instability, *LS* Limb Salvage, *HHS* Harries Hip Score, *MSTS* Musculoskeletal Tumor Society

Previously, the average MSTS score of APCs reported in literatures ranged from 58.3 to 92.0%, while the average HHS from 67.6 to 90.3 [1, 11, 13, 15, 18, 20, 21, 23, 29, 30, 32]. Favorable functional outcomes were demonstrated in our study. The average MSTS score and HHS were 80% (33.3–100%) and 83 (48–100) respectively, which were comparable with other studies [1, 13, 31]. Although the MSTS score and HHS at the last follow-up decreased, no statistical significance was found when compared with that at one-year follow-up (*P* = 0.7 and *P* = 0.74). In our opinion, effective reconstruction of the abductors’ attachment, especially the gluteus medius, is the key factor for the good function [15, 30, 33].

The failure rate for APC ranged from 10 to 50% [9]. The aseptic loosening, structure failure, infection and local recurrence contribute to the major causes for revisions [9]. In our study, the fracture was the most frequent complication including 5 intraoperative periprosthetic crack fractures, 2 PPPFs and 2 trochanteric fractures. This might have a great relationship with the fixation way of the prosthesis in the bone. Compared to cemented APC, the uncemented reconstruction was more skillful because the allograft and host bone medullary canal had to be reamed appropriately to avoid the early stem loosening and the periprosthetic fracture. Our intraoperative crack fractures were fixed with cerclage wires, the axial and torsional stabilities should be ensured, otherwise, plate fixation should be used. For comminuted fractures, a new allograft or extra plate fixation would be used if the stability was not achieved with cerclage wires [34]. Additionally, biomechanical test showed the fixation was stable enough even the longitudinal femoral crack fracture extended 4 cm distal to the lesser trochanter, compared with cable, or, a combination of cable and plate [35]. This secure fixation makes extra rehabilitation programs unnecessary. Two transverse PPPFs occurred 10 mm and 37 mm above the allograft-host junction line respectively due to powerful trauma, which indirectly verified the bone union at the allograft-host junction. The revision standard depends on the quality of the allograft. If the quality of the allograft is poor, endoprosthesis revision should be appropriate, or else, allograft should be preserved. In other literature, seven (18.4%) periprosthetic fractures have been reported when using APC with short cemented stem and compressing plate [18]. Therefore, the long enough stem fixation demonstrates better load-sharing. And, the ratio of prosthetic length in host and allograft bone is recommended close to (1–1.5):1. In our study, two patients have been observed the trochanteric fracture with excellent and asymptomatic function during follow-up. The reason may be related to a strong fibrosis tissue formed by the periarticular soft tissues [1].

The bone union at the allograft-host junction seems regarded as one of the most important advantages of APC [1, 15, 16]. Theoretically, without the cement block at the allograft-host junction, the bone union could be achieved internally and externally [36]. Thus, from 2007, uncemented prosthesis was applied in our institute [23]. The results showed all patients achieved on-time bone union with the average union time of 7.6 months, which was shorter than most studies but comparable to Donati et al. and Malhotra et al. [13, 19–21, 30]. In addition, the nonunion rate at the allograft-host junction of our present study was much lower than previous studies including ours [20, 21, 30, 32, 37]. This may be related to the granular allogenous bone grafting, the preservation of a 10-15 mm normal periosteal cuff, the initial stability between the stem and both the allograft and the host bone, and 4 weeks of bed rest after operation [23]. In our study, only 5 malignant patients received chemotherapy. Although chemotherapy could influence the union of the allograft-host junction, such a small proportion of chemotherapeutic patients in our series cannot confirm the relationship [38]. To minimize the nonunion rate at the allograft-host junction of cemented APC, short cemented stem with compressing plate has been introduced [18]. However, its own disadvantages cannot be avoided, such as extra destroying to the periosteum and related muscle attachments, higher rate of PPPF, longer operative duration, longer incision and higher cost.

LR is another concern. The revision rate for tumor recurrence can be as high as 11% on endoprosthesis reconstruction, while 5% on APC [15, 39]. One possible reason for lower recurrence on APC is the immunogenicity of damage-associated molecular patterns (DAMPs), which are triggers of allograft rejection following release from dying cells. These strong triggers of immune response will give rise to the success of cancer immunotherapy [40]. However, in our cohort, 3 patients (16.7%) occurred LR, which was much higher than that of previous reports using endoprosthesis and APC reconstruction [9]. The potential reason is that among these 3 patients, 1 had previous surgery and pathological fracture while another 2 had a previous surgery before. This phenomenon has been discussed before [41].

The allograft resorption has been reported as high as 97.8% and usually occurred in Gruen zone 1 and 7 [1, 21, 23], while one study also showed Gruen zone 2 and 3 were the most affected areas [29]. In our series, the Gruen zone 1 and 7 were the most ordinary and severe affected zones, of which half was combined with moderate resorption in the Gruen zone 2. Three reasons may lead to the high rate of the allograft resorption. First, the surrounding tissue of the allograft composes of chronic inflammatory cells, histiocytes and foreign-body giant cells, which will active osteoclastic resorption [36]. Second, Gruen 1 and Gruen 7 are known as the tensile stress zone. The stress centralized in these areas after iliopsoas and gluteus medius reattachment may cause the resorption [23]. Finally, the allograft remodeling consists of 3 consecutive phases, resorption, reversal and formation [42]. Interestingly, no statistical significance was observed with regard to function between patients who developed resorption or not (*P* = 0.082). From our viewpoints, the reason may be related to a strong fibrous tissue shaped by the periarticular soft tissues and inserted on the composite allograft [1]. No treatments were taken for these patients as there was no obvious radiolucent line between the allograft and the prosthesis, no subsidence of the prosthesis in the allograft and asymptomatic.

Our study has some limitations. On one hand, our study was limited with its retrospective design with no control group. On the other hand, the sample in this study was small. Although our study was the largest uncemented APC sample reconstruction for neoplastic defect of the proximal femur, only 18 patients with a minimal 65 months follow-up were included. A multi-center with more samples were needed.

## Conclusion

Uncemented APC holds the merits of shortening bone union time at the allograft-host junction and fewer nonunion. Based on our previous short-term outcome, long-term clinical results further verify that it is a reliable reconstruction. With proper procedures and postoperative management, patients can achieve good results in the long-term follow-up, particularly for those who were expected for long living with a good function after resection of the proximal femoral bone neoplasm. Although some complications still exist, the resorption and trochanteric fracture seem to have no effect on the stability of the composite and the function.


**Additional file 2**

## Supplementary Information

**Additional file 1.**

## Data Availability

The datasets used and analyzed during the current study are available from the corresponding author on reasonable request.

## Abbreviations

APC: Allograft prosthesis composite
RHA: Revision hip arthroplasty
CS: Chondrosarcoma
MFH: Malignant fibrous histiocytoma
GCTB: Giant cell tumor of bone
OB: Osteoblastoma
OBOS: Osteoblastic osteosarcoma
OS: Osteosarcoma
FBOS: Fibroblastic osteosarcoma
AJCC: American Joint Committee on Cancer
CT: Computed tomography
MRI: Magnetic resonance imaging
SPECT: Single-photon emission computed tomography
HHS: Harris Hip Score
MSTS: Musculoskeletal Tumor Society
AP: Anterior-posterior
T-SMART: Tomosynthesis-Shimadzu metal artifact reduction technology
PPPF: Postoperative periprosthetic and prosthetic fracture
LR: Local recurrence
THA: Total hip arthroplasty
DAMPs: Damage-associated molecular patterns
